# Contribution of electrical impedance tomography to personalize positive end-expiratory pressure under ECCO_2_R

**DOI:** 10.1186/s13054-024-04908-0

**Published:** 2024-04-16

**Authors:** Benjamin Pequignot, Alain Combes, Mickael Lescroart, Bruno Levy, Matthieu Koszutski

**Affiliations:** 1 Service de Médecine Intensive et Réanimation, Université de Lorraine, Hôpital Brabois, CHRU Nancy, 54500 Vandoeuvre Les Nancy, France; 2 Faculté de Médecine de Nancy, Université de Lorraine, INSERM UMR_S1116, Vandœuvre-Lès-Nancy, France; 3 Institute of Cardiometabolism and Nutrition, Sorbonne Université, INSERM, UMRS_1166-ICAN, 47, Boulevard de L’Hôpital, 75013 Paris, France; 4 Service de Médecine Intensive-Réanimation, Institut de Cardiologie, APHP Hôpital Pitié–Salpêtrière, 75013 Paris, France

**Keywords:** ARDS, ECCO2R, EIT, Mechanical ventilation

## Abstract

Extracorporeal Carbon Dioxide Removal (ECCO_2_R) is used in acute respiratory distress syndrome (ARDS) patients to facilitate lung-protective ventilatory strategies. Electrical Impedance Tomography (EIT) allows individual, non-invasive, real-time, bedside, radiation-free imaging of the lungs, providing global and regional dynamic lung analyses. To provide new insights for future ECCO2R research in ARDS, we propose a potential application of EIT to personalize End-Expiratory Pressure (PEEP) following each reduction in tidal volume (VT), as demonstrated in an illustrative case. A 72-year-old male with COVID-19 was admitted to the ICU for moderate ARDS. Monitoring with EIT was started to determine the optimal PEEP value (PEEP_EIT_), defined as the intersection of the collapse and overdistention curves, after each reduction in VT during ECCO_2_R. The identified PEEP_EIT_ values were notably low (< 10 cmH2O). The decrease in VT associated with PEEP_EIT_ levels resulted in improved lung compliance, reduced driving pressure and a more uniform ventilation pattern. Despite current Randomized Controlled Trials showing that ultra-protective ventilation with ECCO_2_R does not improve survival, the applicability of universal ultra-protective ventilation settings for all patients remains a subject of debate. Inappropriately set PEEP levels can lead to alveolar collapse or overdistension, potentially negating the benefits of VT reduction. EIT facilitates real-time monitoring of derecruitment associated with VT reduction, guiding physicians in determining the optimal PEEP value after each decrease in tidal volume. This original description of using EIT under ECCO_2_R to adjust PEEP at a level compromising between recruitability and overdistention could be a crucial element for future research on ECCO_2_R.

To the Editor

Extracorporeal carbon dioxide removal (ECCO_2_R) is a device designed to eliminate carbon dioxide in patients with acute respiratory failure. In acute respiratory distress syndrome (ARDS), ECCO_2_R facilitates lung-protective ventilatory strategies, by allowing a reduction of tidal volumes and thus generating less ventilator-induced lung injuries (VILI) [1]. Despite the limited availability of high-quality evidence, the utilization of ECCO_2_R is increasingly prevalent [2–4]. In ARDS under Extracorporeal life support (ECLS), the exact safe limits for volume and pressure settings remain unclear. Evidence suggests that deviating from conventional lung-protective ventilation, involving a reduction in the intensity of mechanical ventilation, may be associated with improved outcomes [5]. Ultraprotective ventilation with low tidal volumes may inadvertently cause pulmonary derecruitment if positive end-expiratory pressure (PEEP) is not adequately titrated. Conversely, inappropriately high PEEP levels may lead to overdistention [6]. Regarding PEEP and ECCO_2_R, in the SUPERNOVA pilot trial, which investigated the feasibility and safety of three different ECCO_2_R systems in ARDS patients, PEEP was titrated to target a plateau pressure (P_PLAT_) of 23–25 cmH_2_O, yielding a median value of 15.5 [10.0–16.0] cmH_2_O. In the recently completed REST trial, which evaluated the impact of ECCO_2_R on mortality in ARDS, PEEP was based on ARDSNet tables, resulting in a mean value of 11.3 (± 3.1) cmH_2_O [7].

Electrical impedance tomography (EIT) allows individual, non-invasive, real-time, bedside, radiation-free imaging of the lungs, providing global and regional dynamic lung analyses. Recent study results underscore the utility of EIT in identifying the optimal PEEP during venovenous extracorporeal membrane oxygenation with low tidal volume [8]. To provide new insights for future ECCO2R research in ARDS and to avoid a "one size fits all" approach we present a potential application of EIT to personalize PEEP selection under ECCO2R, following each decrease in tidal volume.

## Patient

A 72-year-old male with no prior respiratory history was intubated due to SARS-CoV-2-related moderate ARDS. His respiratory mechanics progressively worsened, with a respiratory system compliance (C_RS_) of 22 mL/cmH_2_O, a driving pressure (ΔP) of 19 cmH_2_O and a tidal volume of 420 mL at a PEEP level of 11 cmH_2_O. ECCO_2_R was initiated to enhance ultra-protective lung ventilation.

## Assessment

**Fig. 1:**
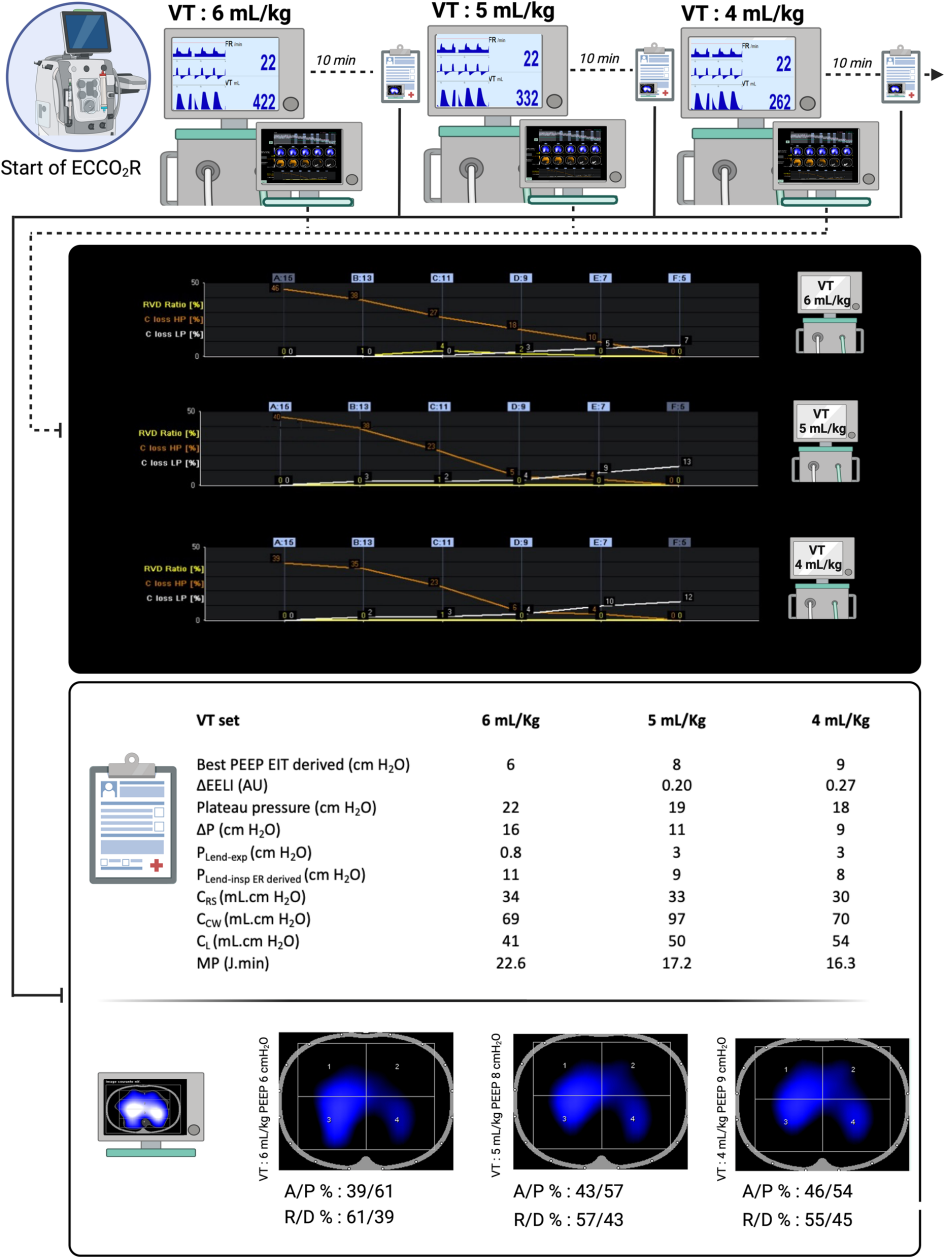
(from top to bottom): decremental PEEP trials from 15 cmH_2_O to 5 cmH_2_O (by steps of 2 cmH_2_O), at each level of tidal volume (VT). Clinical, respiratory and EIT parameters at VT of 6, 5 and 4 ml/kg of PBW with PEEP set at the best EIT-derived PEEP. The last insert represents percentages of variation in impedance during ventilation (ΔZ) in the right (R) and left (L) half of the lung, and anterior (A) and posterior (P) half of the lung. Δ EELI: end-expiratory lung impedance variation. ECCO_2_R: Extracorporeal carbon dioxide removal. P_PLAT_: plateau pressure. ΔP: driving pressure defined as the difference between plateau pressure and total PEEP. P_Lend-insp ER derived_: transpulmonary pressure at end inspiration, computed as follows: P_PLAT_ x (E_L_/E_rs_) where E_L_ is the lung elastance and E_rs_ the respiratory system elastance; P_Lend-exp_: transpulmonary pressure at end expiration C_RS_: respiratory system compliance. C_L_: lung compliance. C_cw_: Chest wall compliance. MP: Mechanical power. EIT: electrical impedance tomography. RVD: Regional ventilatory delay. C loss HP: Compliance loss at high pressure (i.e. overdistention). C loss LP: Compliance loss at low pressure (i.e. collapse)

ECCO_2_R was initiated as part of the PRISMALUNG study (BXU542357). The patient was deeply sedated and paralyzed under assist-control volumetric ventilation. The tidal volume was adjusted to target 6, 5 and 4 mL/Kg of predicted body weight (PBW), each step lasting 10 min. The ‘best’ positive end-expiratory pressure was determined for each tidal volume levels, by EIT during a decremental PEEP trial from 15 cmH_2_O to 5 cmH_2_O, with steps of 2 cmH_2_O for a duration of 2 min [9]. Bedside parameters are derived from the calculus of relative changes in pixel compliance and estimate alveolar collapse and hyperdistension according to the Costa algorithm [10]. We assessed respiratory parameters with esophageal pressure and ventilation distribution with EIT (Dräger, Pulmovista, Lübeck, Germany) at the end of a stabilization period of 10 min after each reduction of tidal volume at the ‘best’ PEEP derived from EIT (PEEP_EIT_) (Fig. 1). Ventilator data were continuously recorded by the EIT-device through a serial interface (Medibus, Dräger Medical, Lübeck, Germany) from the ventilator (Dräger, C500, Lübeck, Germany). The blood flow of ECCO_2_R was gradually set at 400 mL/min without gas flow. Gaz flow was started as soon as PaCO_2_ was > 50 mmHg.

## Results

The optimal PEEP_EIT_, determined after each PEEP trial after reduction of tidal volume was relatively low (< 10 cmH_2_O). The decrease in tidal volume associated with PEEP_EIT_ level resulted in three outcomes:

- First, an improvement in lung compliance (C_L_) associated with a slight increase in end-expiratory lung impedance (EELI).
- Secondly, as expected with ECCO_2_R, a reduction in ΔP and a decrease in the intensity of mechanical ventilation, assessed by mechanical power (MP) calculated according to the equation proposed by Gattinoni et al. [11].
- To finish, this approach led to a more homogeneous ventilation pattern, as indicated by a shift in the ratio between anterior and posterior lung zones from 39/61 to 46/54 between tidal volumes of 6 and 4 mL/Kg.

It is noteworthy that there was no difference in oxygenation at different levels of tidal volume, and we did not observe any hemodynamic events.

## Discussion

The reduction of tidal volume in ARDS, facilitated by ECCO_2_R, primarily aims to decrease VILI. Ultra-protective ventilation supported by extra-corporeal devices failed to improve survival in current RCTs [7]. Whether universal ultra-protective ventilation settings can be applied and benefit to all patients remains controversial. A bundle of tidal volume, I/E ratio, respiratory rate and PEEP level should be daily adjusted to reduce both baro- and atelec-trauma. An inappropriate PEEP level can lead to alveolar collapse or overdistension, and potentially negate the benefits of tidal volume reduction. EIT allows real-time monitoring of the derecruitment associated with the decrease of tidal volume and guides the physician with determining the optimal PEEP value after each reduction in tidal volume. This value was ultimately lower compared to PEEP proposed in guidelines and clinical trials [1, 2, 7]. In the present issue, EIT allowed the selection of personalized PEEP levels accross different tidal volume settings, balancing recruitability and overdistention, maintaining a reduction in mechanical ventilation intensity, and resulting in a more homogeneous distribution of mechanical ventilation.

Our report has also limitations. First, given the short stabilization period between the PEEP trials and serial measurements, each PEEP trial could have had an impact on the lung recruitment, although the highest PEEP was relatively low (15 cmH2O) if we consider it as a recruitment maneuver. Second, the assessment of tidal recruitability was not performed in this patient and may have underestimate the effect of tidal recruitment impacted by tidal volume lowering [12]. Thirdly, the PEEP level achieved using the crossing point strategy is not directly measured by the EIT device, but rather inferred by our team based on the interpolation of overdistention and collapse curves. Additionally, the determined PEEP level from the crossing point is influenced by the range of PEEP settings. This variability can be attributed to the computational method employed for assessing lung collapse and overdistension, which involves comparing maximal compliance with current compliance for each pixel at a specified PEEP level. At lower PEEP levels, overdistension is consistently calculated as zero due to the algorithm’s design, although it is conceivable that overdistention may not truly be absent. The crossing point would have been perhaps different with other ranges of PEEP during the PEEP trial. Although PEEP_EIT_ tends to reduce both overdistension and collapse, this does not necessarily mean that this PEEP level will be the one that best mitigates VILI despite a more homogenous ventilation [13, 14].

## Conclusion

During tidal volume reduction under ECCO_2_R, EIT allows the personalization of the PEEP level by allowing a bedside evaluation of the compromise between recruitability and overdistention, even if the EIT based technique is still not free from uncertainties. This original description presents a strategy avoiding the "one-size-fits-all" approach to mechanical ventilation in ARDS patients, and suggests potential future aspects of research on ARDS and ECCO_2_R.

## Data Availability

The data used and/or analysed during the current assessment are available from the corresponding author on reasonable request.

## Abbreviations

ECCO_2_R: Extracorporeal carbon dioxide removal
ARDS: Acute respiratory distress syndrome
VILI: Ventilator-induced lung injury
PBW: Predicted body weight
P_PLAT_: Plateau pressure
C_RS_: Respiratory system compliance
C_L_: Lung compliance
C_CW_: Chest wall compliance
ΔP: Driving pressure
PEEP_EIT_: ‘Best’ PEEP derived from EIT
EELV: End-expiratory lung volume
MP: Mechanical Power
VT: Tidal volume
